# A Piezoelectric Plethysmograph Sensor Based on a Pt Wire Implanted Lead Lanthanum Zirconate Titanate Bulk Ceramic

**DOI:** 10.3390/s100807146

**Published:** 2010-07-29

**Authors:** Carlos O. González-Morán, J.J. Agustín Flores-Cuautle, Ernesto Suaste-Gómez

**Affiliations:** Centro de Investigación y de Estudios Avanzados del IPN, Electrical Engineering Department, Bioelectronics Section. Av. IPN 2508, Col. San Pedro Zacatenco, C.P. 07360, México D.F., Mexico; E-Mails: cgonle@cinvestav.mx (C.O.G.-M.); jjflores@cinvestav.mx (J.J.A.F.-C.)

**Keywords:** PLZT application, ceramic implants, plethysmography

## Abstract

This work reports on the development of a Lead Lanthanum Zirconate Titanate (PLZT) bulk ferroelectric poled ceramic structure as a Piezoelectric Plethysmograph (PZPG) sensor. The ceramic was implanted during its fabrication with a platinum (Pt) wire which works as an internal electrode. The ceramic was then submitted to an experimental setup in order to validate and determine the Pt-wire mechanical effects. This PZPG sensor was also mounted on a finger splint in order to measure the blood flow that results from the pulsations of blood occurring with each heartbeat. Fingertip pulses were recorded jointly with an ECG signal from a 25 year old male to compare the time shift; the PZPG sensor guarantees the electrical isolation of the patient. The proposed PZPG has several advantages: it can be adjusted for fingertip measurements, but it can easily be extended by means of spare bands, therefore making possible PZPG measurements from different body locations, e.g., forehead, forearm, knee, neck, *etc*.

## 1. Introduction

**Figure 1.**
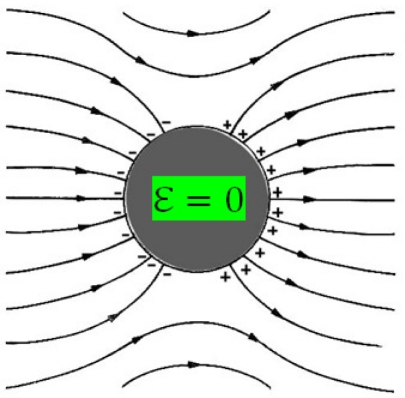
Electric field waves across Pt-wire (top view).

Among piezoelectric materials, Lead Lanthanum Zirconate Titanate (PLZT) is one of the most popular. PLZT is a ferroelectric solid solution with wide-ranging material properties that depend on its composition [1]. Recently, a new PLZT bulk single plate, called ceramic-controlled piezoelectric (CCP) has been produced; this CCP has a 300 μm Pt-wire implant in order to form a free upper face that measures optic and mechanic events [2]. This structure design includes a modified electrode configuration: a Pt-wire introduced into a bulk PLZT. The Pt-wire was chosen as an implant because it possesses high resistance to chemical attack, it has excellent high-temperature characteristics (melting point 1,768.3 °C), and it has stable electrical properties and thermal conductivity with small variations [3]. When CCP is polarized, the mobile charges in the Pt-wire accumulate on its surface until the field produced completely cancels the external field applied to the conductor; this results in electrical equilibrium within the Pt-wire so the internal the electrical field is zero, as shown in Figure 1. This means that the net charge is distributed over a section of the surface, which has a thickness of several atomic layers, but not in the geometrical sense [4]. The sample preparation is described in the following sections [2].

The Pt-wire cylinder dimensions are 10 mm high and 0.3 mm diameter; this gives a surface area of 9.42 mm^2^. The surface area of one of the faces of the ceramic is 78.53 mm^2^, making the surface area of the ceramic 8.33 times bigger than that of the Pt-wire.

**Figure 2.**
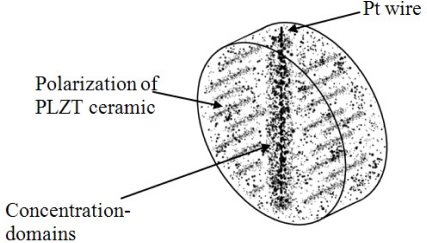
Scheme of the CCP and its domains distributed around the Pt-wire.

Pt-wire implanted into bulk PLZT ceramic behaves singularly in the CCP due to the domains coating the Pt-wire, as shown in Figure 2. This type of domain wall separates domains polarized perpendicularly to each other and when the CCP is polarized, the field should be such as to exert opposite torques on the polar axes of opposite domains, but not large enough as to permanently rotate the polarization. A similar effect is obtained by applying a suitable stress to the ceramic, as the piezoelectric effect has different signs in antiparallel domains.

When the CCP is poled, it originates a great concentration of domains on the Pt-wire because the dipoles are oriented over all its external part. These concentrations achieve free flux charges around the Pt-wire when CCP is excited by stress on its side face.

Considering that ferroelectric and piezoelectric materials can be used as sensors and actuators, piezoelectric pressure and acceleration sensors, as well as a variety of piezo-vibrators, are now commercially available. The development of ultrasonic motors for a variety of new applications has been dramatic and widespread in recent years. Among piezoelectric ceramics, CCP offers a great variety of applications in medical physics such as the human pulse detection sensor known as PZPG.

In medical physics, micro-circulation of skin blood is a subject of considerable interest due to its role in human metabolism, blood transport from and to the tissue and its role as a liquid coolant of the body in the thermoregulation process.

There are several techniques which are used to follow the blood flow in living tissues [5]. Piezoelectric methods seem to be the most promising for skin microcirculation studies; one advantage of piezoelectric sensors is that they can be used for true dynamic measurements due their wide range of frequency operation. Therefore, the analysis of the skin mechanic pulse piezoelectric detection provides valuable selective information on blood flow on upper skin layers, cutting off the influence of the deeper arteries and veins [6].

The non-invasive piezoelectric method uses the mechanical signals for temporal analysis of the skin blood volume pulsations. The periodical increase of blood volume in micro-vessels due to their dilatation during the systolic raise of pressure with the following diastolic contraction over each heartbeat causes corresponding changes in the absorption of the mechanical signals which travel within the working volume.

The measurement of the blood flow is related to the measurement of changes in volume which occur in any part of the body as result from the pulsations of blood with each heartbeat. The instruments that measure volume changes or provide outputs that can be related to them are called plethysmographs. Plethysmographs respond to changes in volume, but there are several devices that in fact measure some other variables related to volume rather than volume itself. One type of these “pseudo-plethysmographs” measures changes in diameter at a certain cross section of a finger, toe, arm, leg or other segment of the body, for example, the non-invasive reflection photoelectric plethysmograph method uses back-scattered optical signals for temporal analysis of skin blood volume pulsations [7].

The pseudo-plethysmograph techniques have been substantially improved since their origins, thanks to the development of a new PLZT bulk ceramic structure design [2,8,9]. This improvement has opened new horizons for the implementation of this method in clinical praxis, self-monitoring and tele-medicine. Besides this possible applications, this pseudo-plethysmograph based on PLZT guarantees electrical isolation of the patient.

The objective of this work was to explain the effect of the Pt-wire implanted into a PLZT ceramic bulk and validate it in a mechanical experimental setup. Then the heartbeat pulses from a human being detected by this CCP were recorded in an experimental setup. This development also demonstrated the enormous relevance of measuring cardiac pulses at a cross section of the index finger.

## 2. Methodology

A ferroelectric PLZT was chosen; this is a Pb_1-x_La_x_(Zr_1-y_Ti_y_)_1-x/4_O_3_ ceramic with x = 0.09 and y = 0.35 (PLZT), generally denoted as (9/65/35). This ceramic was produced by the oxide-mixing technique: the raw materials were mixed by ball-milling with an electronic mill (Pulverisette 2, Fritsch) for 20 min; polyvinyl alcohol drops were added with a rate of 1.5 drops per gram of mixture. Powders then were pressed into discs of 10 mm diameter and 2 mm of thickness; the pressure applied was 3,500 Kg/cm^2^.

During this process, a Pt-wire of 0.3 mm diameter was implanted in the middle of the ceramic in a transversal way; thus a metallic electrode totally immersed in the ceramic was created. This ceramic was sintered in air with a heater ramp rate of 5 °C/min from room temperature to 600 °C and a second heater ramp rate of 10 °C/min from 600 °C to 1,200 °C; the latter process lasted for one hour in a platinum crucible.

After sinterization, silver electrodes were deposited on the lower face and the Pt-wire of the CCP. Finally the discs were electrically poled, at 1.5 kV/mm for one hour at 60 °C in a silicone oil bath, in order to be used in pulse measurements. The dielectric constant was determined by the capacitance measure. Samples were heated at a rate of 5 °C/min, until 450 °C while the capacitance was measured at 1 kHz with a Beckman LM22A RLC bridge. The dielectric constant was determined by expression (1):

(1) $$ɛ=\;\mathrm{Cl}/{ɛ}_{0}A$$

where *C* is the capacitance in *F*, *l* is the thickness of the sample in *m*, *A* the sample area in *m*^2^ and ɛ_0_ the vacuum permittivity = 8.85 × 10−12 *F/m*. The dielectric constant in the CCP was determined with and without the Pt-wire taking the parallel sides as electrodes.

**Figure 3.**
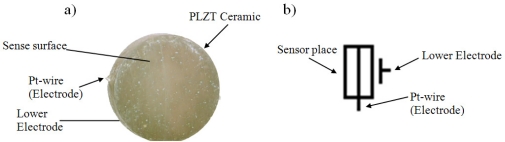
PZPG and its configuration, **(a)** Schematic PZPG implanted with Pt wire, **(b)** Graphical Symbol of PZPG.

**Figure 4.**
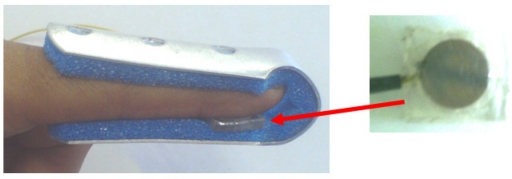
PZPG sensor mounted internally on an index finger splint.

Usually electrodes are tacking at parallel sides and Pt-wire is the third electrode however for this application it will be use one parallel side and Pt-wire as electrodes. The schematic symbol b) used for describing the PZPG and the graphical symbol can be seen in Figure 3. The PZPG sensor was placed in a finger splint for cardiac signal detection as shown in Figure 4.

## 3. Experimental Setups

**Figure 5.**
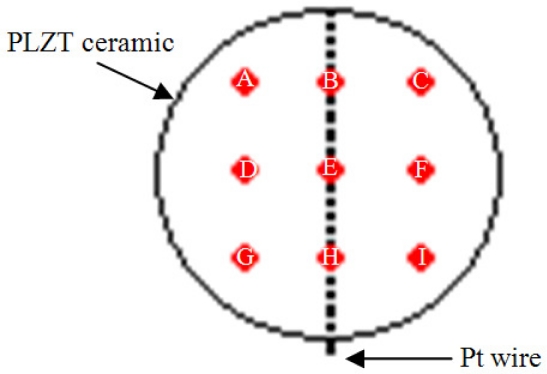
Zones selected for the mechanical analysis.

In order to validate and determine the sensible characteristics of the CCP, experimental mechanical and optical setups were used. Six specific zones were selected at random on a free side face of the CCP in order to detect the mechanical signals (mechanical case), Figure 5.

### 3.1. Mechanical Setup

**Figure 6.**
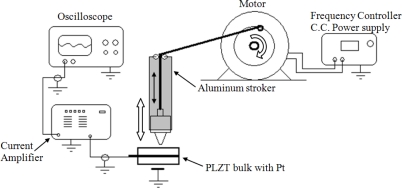
Mechanical setup to get the sensibility on the CCP selected zones.

This setup consisted of constant weight pulses (gram-force) at a frequency of 2.26 Hz on the CCP on the same zones that were used for the optical setup. The components of this setup were a 24 V CD Micro Switch 33VM82-020-11control motor, a SR570 SRS-Low Current Noise Preamplifier and an Agilent DSO3062A oscilloscope. The CCP was struck in a pulsed form controlled by a purposely developed mechanism (Figure 6).

The force applied was 53.9 mN and the signals were recorded with the preamplifier and registered by an oscilloscope which measured the peak to peak voltage in order to create the corresponding graphics showing the magnitude of the direct piezoelectric effect of each record.

### 3.2. Optical Setup

**Figure 7.**
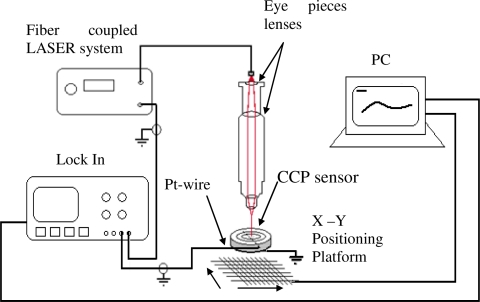
Optical setup to get the sensibility on the selected CCP zones.

In this case we included a set of lenses to reduce the laser beam spot to a 50 μm diameter size. The CCP was placed on an X-Y translation microscope stage controlled by a PC, with a resolution of 50 μm, driven by stepping motors. The analysis of the CCP was done in a zone of 5 mm by 6 mm of a total of 750 points records, as shown in Figure 7. The electrical contacts from the CCP were connected to a DSP Lock-in amplifier to obtain 2D scans; those signals were processed by a computer program measuring their magnitude and phase to construct the corresponding 3D graph whose Z-axis was the photovoltaic current (pA) due its photovoltaic response.

**Figure 8.**
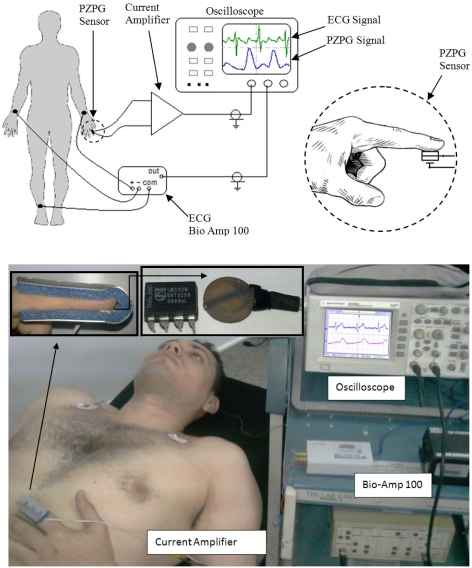
Experimental setup to obtain ECG and PZPG signals from human beings, left: schematic setup, right: real setup.

The experimental setup used in order to obtain the patient’s cardiac pulses was as follows: he was placed in a supine position and in order to record the electrical activity of his heart, the electrocardiographic (ECG) electrodes were placed on the lead I. The ECG signal was measured with an isolated Bio Amp 100 by Axon Instruments; this ECG signal was used for educational purposes: later it was compared with the pulse obtained from PZPG. The PZPG was placed on the index finger of the patients’ left arm, as shown in Figure 8.

The electrical contacts (electrode, Pt-wire) of PZPG were connected to an SR570 SRS-Low Current Noise Preamplifier amplifier in order to obtain a piezoelectric response; the ECG and PZPG signals were registered in the oscilloscope, which was used as a cardiac monitor and to measure the peak to peak voltage.

## 4. Results

**Figure 9.**
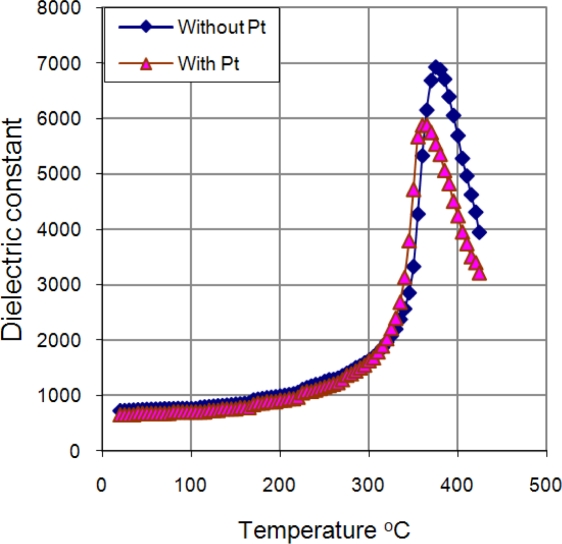
Dielectric constant of PLZT ceramic with and without Pt-wire.

Studies of the dielectric constant ɛ as function of temperature in PLZT ceramic samples (with and without Pt-wire) were carried out. Figure 9 shows the dependence of the dielectric constant with the temperature. The graphic shows that ferroelectric phase transition T_c_ had a shift from 375 °C to 360 °C and a fall of ɛ from 6,923 to 5,889 with Pt-wire.

**Figure 10.**
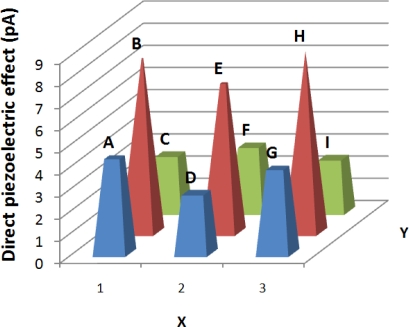
Electrical current generated by a force of 53 mN in six zones.

**Figure 11.**
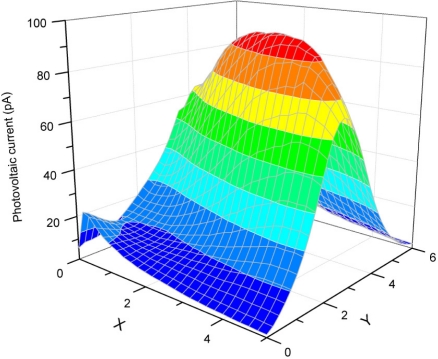
Photovoltaic current generated by a 160 mW/cm^2^ of LASER illumination in 750 dots.

The direct piezoelectric effect was evaluated on selected zones of the CCP; the magnitude of each zone was different, as shown in Figure 10. However, an important rise was observed on the Pt-wire zone (B, E, H zone). It was clearly seen that the signal average magnitudes from zones B, E and H were 42.79 % bigger than in the no Pt-wire zones. Figure 11 shows the photovoltaic current (pA) due its photovoltaic response.

**Figure 12.**
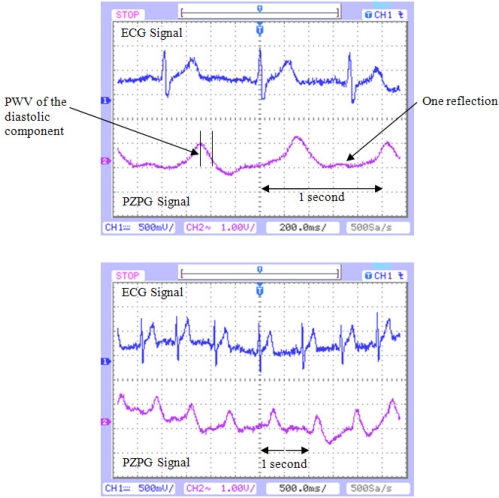
ECG and PZPG signal from a 25 year old man. It shows the pulse wave velocity and the reflections

Many PZPG studies using the fingertip as the measurement site have been performed. The fingertip PZPG signals are relatively strong, thanks to its anatomical feature of a large vascular bed and, consequently, the presence of an important total pulsating blood volume. The basic design of the device was relatively simple. The PZPG showed satisfactory results due to the signal response, in the cardiac monitor graphics ECG and PZPG detection that provides additional diagnostic information on the vascular blood flow resistance; the cardiac pulses can be obtained by measuring the heartbeat wave propagation time in fingertip site as the time shift between the two corresponding signals ECG and PZPG pulses. The height of the diastolic component of the PZPG relates to the amount of the pressure wave reflection, which relates mainly to the tone of small arteries. The timing of the diastolic component relative to the systolic component depends on the pulse wave velocity (PWV) of the pressure waves in the aorta and the large arteries. This, in turn, depends on the large artery stiffness. The human vascular system is elastic and multi-branched, and each branching partially reflects back the pressure wave [10]. Figure 12 shows ECG and PZPG signals on an oscilloscope. These signals represent a measurement taken from a healthy male young man and they only demonstrated the functionality of the PZPG. The T wave of the ECG complex was shifted 80 ms; the patient’s cardiac pulse which was detected at 360 ms was identified as a reflection.

## 5. Discussion

The PZPG device demonstrated a good performance in optic and mechanic probes as an opacity sensor [11] and cardiac pulse measurement device, respectively. A study with several people implies a research protocol in order to determine the study basis such as blood pressure detection, cardiac pulse in different zones of the body, continuous cardiac pulse, and the associated cardiac variability with electrocardiogram data. The legal steps in order to carry out these studies with patients of several ages are pending.

The CCP has an interesting configuration because by the one hand it provides a great ceramic insulator that does not affect human beings and on the other hand it is very significant that this sensor was developed not only for this mechanical field of study, but also has multiple applications in different areas, for example: optics, acoustics, electrical and chemical sensing. Finally is important to mention that an advantage of an implanted ceramic over a conventional ceramic (with two parallel conductive layers) is the increase of a mechanical and optical signal measured due the physical reduction (cutting the piece) making minisensors for multiple uses such as neonatal applications.

## 6. Conclusions

On the one hand, these results demonstrated that the PLZT bulk with Pt-wire has the following advantages: the increase in surface analysis is superior due to the Pt-wire, which works as a third electrode; the proposed CCP can be used at much higher temperatures than the conventional Si based sensors; the CCP is easy to make and the size of this bulk material can be modified; finally, the CCP offers good versatility as a mechanical sensor due to its ferroelectricity.

By means of mechanical and optical probes of PZPG it is possible to see the implant effect, since as shown in Figures 10 and 11, the region near the implant has a high domains concentration and these concentrations can be detected optical and mechanically, to the contrary, the parallel plate PLZT response is flat, when PZT parallel plate and PZPG are compared the PZPG shows a superior response in the area near to the implant as high as 42%.

On the other hand, it was shown that the PZPG has several advantages. It can be adjusted to the fingertip measurements; however, it can also easily be extended by means of spare bands, therefore it is possible to take PZPG measurements from different locations of the body, e.g., forehead, forearm, knee, neck. It is non invasive due to its mechanical detection and there are no chemical reactions with the body or possible current discharges because it is completely isolated and it does not require external electrical supply. The implant is put into the ceramic during the sintering stage, meaning that no additional step is required, whereas coating the ceramic with a polymer or any other material involves one or more fabrication and characterization steps, which in an industrial process is more expensive. Moreover, this setup eliminates considerable electrical noise.

The timing of the diastolic component and the reflections, according to the place of measure, can be determined with the PZPG. The next step is to develop the interface and software to connect the PZPG sensor to any computer in order to make many recordings at different body locations.
